# A Brief Overview on Epoxies in Electronics: Properties, Applications, and Modifications

**DOI:** 10.3390/polym15193964

**Published:** 2023-09-30

**Authors:** Rashid Dallaev, Tatiana Pisarenko, Nikola Papež, Petr Sadovský, Vladimír Holcman

**Affiliations:** Department of Physics, Faculty of Electrical Engineering and Communication, Brno University of Technology, Technická 2848/8, 61600 Brno, Czech Republic; 177722@vut.cz (T.P.); papez@vut.cz (N.P.); holcman@vut.cz (V.H.)

**Keywords:** epoxy resins, thermal conductivity, dielectric strength, chemical modifications, electrical conductivity, curing

## Abstract

This paper offers a short overview of epoxy resins, encompassing their diverse characteristics, variants, chemical modifications, curing processes, and intriguing electrical properties. Epoxies, valued for their multifunctional attributes, serve as fundamental materials across industries. In the realm of dielectric strength, epoxy resins play a crucial role in electrical insulation. This paper discusses the mechanisms governing dielectric breakdown, strategies to enhance dielectric strength, and the impact of various fillers and additives on insulation performance. Through an exploration of recent research and advancements, this paper delves into the spectrum of epoxy properties, the array of subspecies and variants, their chemical adaptability, and the intricacies of curing. The examination of electrical resistance and conductivity, with a focus on their frequency-dependent behavior, forms a pivotal aspect of the discussion. By shedding light on these dimensions, this review provides a concise yet holistic understanding of epoxies and their role in shaping modern materials science.

## 1. Introduction

### 1.1. Properties of Epoxy Resins

The evolving landscape of electronics and microprocessor technology, characterized by increasingly potent components within devices, necessitates the advancement of highly efficient composite materials. This also calls for the innovation of robust resins (polymeric materials) that are capable of performing reliably under extreme conditions, including elevated temperatures, exposure to solar radiation, and high humidity levels. These materials are essential for effectively sealing electronic devices that are made from diverse substrates such as plastic, brass, aluminum, copper, and carbon steels. Additionally, they play a vital role in providing protective coatings for structural materials, safeguarding them against various forms of impact and shock [1,2].

The range of use is determined by the parameters of epoxy resins (ERs): thermal conductivity and electrical conductivity. Ensuring reliable adhesion on boards formed from fiberglass with plastic and metal, as well as a combination of dielectric properties with light transmission, are in demand in microelectronics and electrical engineering [3].

The widespread use of epoxy resins in the production of electronic components is due to their high manufacturability and the unique set of performance characteristics of their curing products [4,5,6,7]:

- Excellent dielectric/electrical insulating properties;
- Ultra-high water resistance;
- Chemical resistance (resistance to a number of aggressive chemicals, alkalis, acids, salts, solvents);
- High mechanical properties (ideal tensile strength, wear resistance, hardness, impact resistance);
- The highest adhesion (high-quality glue connection);
- Low shrinkage during curing (minor deformations);
- High stability;
- Increased heat resistance (withstands temperatures up to 150–200 °C. High coefficient of thermal conductivity);
- The absence of volatile products and the release of side substances during curing;
- A low coefficient of shrinkage in the course of hardening;
- Minimum creep under load;
- Low odor;
- Ease of production and versatility of processing processes;
- Small weight of the finished product;
- Durability and shelf life;
- Environmental friendliness;
- Safety.

The complex of unique physicomechanical (hardness, elasticity, tensile strength, density, conductivity) and physicochemical properties (solubility, reactivity, pH level, heat of combustion, electronegativity) of epoxy resin allows products based on them to cover a wide range of applications in various fields of electrical and radio engineering. Scientific and technical research that focuses on the development of the most specialized coatings (epoxy fills) that are resistant to highly aggressive environments, improving the properties of epoxy resin as a dielectric (electrical conductivity and insulation strength), and satisfying certain operating conditions is steadily growing [8]. 

At the present stage of the development of space technology, it has been established that the most dangerous factor for existing spacecraft is the functional damage to on-board radio-electronic equipment from ionizing and secondary electromagnetic radiation. The impact of such radiation leads to structural changes in materials, the occurrence of ionization, heating, the appearance of induced radioactivity, and other phenomena that disrupt physical and chemical processes in technical devices [9].

After curing, epoxy resins act as a high-quality insulator that provides the sealing (low hygroscopicity and electrical insulation) of conductive circuits from the effects of electrostatic discharge, high temperatures, vibrations, moisture, chemicals, and shocks in electrical and radio engineering products.

### 1.2. Applications of Epoxy Resins

Epoxy resin hold significant importance within the electronics industry and find applications in a range of equipment including motors, generators, transformers, switchgear, bushings, and insulators. These epoxy resins offer outstanding electrical insulation properties, effectively shielding electrical components from potential issues like short circuits, dust ingress, and moisture exposure [10].

In addition, metal-filled polymers are widely adopted for electromagnetic interference shielding [11]. Epoxy molding compounds (EMCs) are favored as encapsulation materials for semiconductor devices, safeguarding integrated circuit components against environmental challenges such as moisture, mobile-ion contaminants, temperature variations, radiation, and humidity, as well as mechanical and physical damage. Furthermore, epoxy composites, enriched with particulate fillers like fused silica, glass powder, and mineral silica, have seen extensive use as substrate materials in electronic packaging applications [12].

As sealants, epoxy resins (compounds) remain popular among polymeric materials in terms of application. To date, due to the thermodynamic and reactive compatibility of epoxy oligomers, a huge number of compounds have been synthesized, and various hardeners have been successfully used to turn thermoplastic resins into infusible ones [13].

ERs are one of the most important class of thermoplastics and the following broad areas of their application are listed below [14,15,16,17]:

- Radio, electrical industry, electronics, and microelectronics (chip assembly and printed circuit board assembly, which is an epoxy–fiberglass composite);
- The rocket and space industry;
- The energy industry;
- The engineering industry (production of components);
- Construction (paints and varnishes for interior and exterior decoration of buildings; polishes; the production of durable impregnations and structural materials (PCM); sealants; adhesives; the waterproofing of floors, walls of basements, and swimming pools; the impregnation of concrete, brick, wood, and mortar; the successful replacement of bitumen—bitumen–epoxy composition);
- The textile and leather industries;
- The furniture industry (designer furniture);
- Arts and crafts (the production of unique costume jewelry; the production of artificial amber; models; crafts; dioramas; and in bench modeling);
- The domestic sphere and household (home decor; bar counters).

Their implementations, specifically in the production of electronic components, are the following [18,19,20]:

1. Printed circuit boards (PCBs): epoxy resin is used as a substrate material for printed circuit boards, providing insulation between board layers and protecting components from environmental influences;
2. Sealing: epoxy resin is used to seal electronic components, protecting them from environmental influences such as moisture and dust;
3. Adhesives: epoxy resin is used as an adhesive in the production of electronic components, and is a sensitive strong bond between components and substrates;
4. Coatings: epoxy resin is used as a coating material for electronic components, providing protection against moisture, chemical compounds, and abrasion.

### 1.3. Curing and Modification of Epoxy Resins

The use of epoxy resin in the manufacturing of electronic components typically involves a curing process, in which the resin is mixed with hardener and then subjected to heating or ultraviolet radiation to initiate a curing reaction. Amines were among the first epoxy resin hardeners, and they have retained a leading position among all reagents of this type [21,22].

The curing process can be carried out using various technologies, including the following [18]:

- Vacuum impregnation: this involves placing the electronic component within a vacuum chamber, where epoxy resin is drawn into the component under vacuum pressure;
- Pouring: in this method, an electronic component is situated in a mold and epoxy resin is poured over it, effectively sealing the component;
- Dispensing: epoxy is administered onto an electronic component using a dispensing machine;
- Spray coating: here, epoxy resin is applied onto an electronic component via a spray gun.

The cross-linking or curing process is usually slow, especially when small volumes of resin are involved. Fast curing hardeners can be used, but they generate a lot of heat during the process, resulting in a strong exothermic reaction that can damage electronic components and cause high mechanical stress not only on them, but also on the circuit [23].

**Figure 1:**
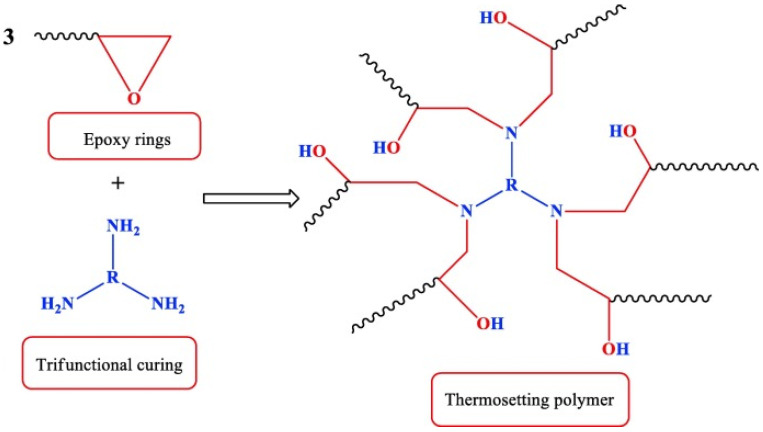
Illustration of the formation process of a polymer with a three-dimensional structure. (Figure from [24], permission granted by “Elsevier”).

The process of curing epoxy polymers involves the utilization of a hardening agent, typically a primary or secondary amine or an acid anhydride. This agent serves as a binding element among the distinct macromolecules. In order to facilitate the creation of a three-dimensional network, which is crucial for conferring the ultimate product with the characteristics of an impenetrable and non-soluble material (as depicted in Figure 1), it is imperative that the hardening agent possesses a functionality of two or greater [24].

Epoxy oligomers and polymers are widely used as matrices for the production of carbon plastics, which are characterized by a combination of high strength and rigidity with low density, a low temperature coefficient of friction, high thermal and electrical conductivity, wear resistance, and resistance to thermal and radiation effects [25].

Epoxy polymers are polar materials, and dipole polarization plays an important role in the dielectric relaxation spectrum. In such polymers, the formation and relaxation of the electrical state is controlled by the interaction of homo- and heterocharges. After polarization, molecular dipoles are oriented in the direction of the polarizing field, and the spatial structure of the polymer is fixed by a three-dimensional network of chemical bonds; as a result, charge carriers are permanently “frozen” in the structure of the network curing product [26].

Studies on the influence of the chemical structure on the properties of epoxy polymers show that a change in the entire complex of polymer properties can be achieved by changing the nature of the initial oligomers and hardeners, as well as their ratio [27].

Epoxy resin is chemically inert; there is a cold, hot, and combined curing. In the liquid state, epoxy resin is extremely vulnerable to high humidity; the cured resin is not toxic.

Now, there are six major classes of epoxy resins—bisphenol (A and F), novolac (phenolic and cresol), aliphatic (mono- and highly functional), glycidyl, and acrylic epoxy. Dozens of their subspecies are known, including light-cured and water-borne ones (their widespread introduction is hindered by high cost) [28].

An integral component of epoxy resin, as a two-component thermosetting polymer, is a hardener, of either the cold or hot type, which determines the further technical characteristics of the epoxy (ductility, strength, hardness, UV resistance, resistance to abrasion). Their percentage ratio is extremely important for the quality performance of the composition. The main part of the additives are the plasticizers: dibutyl phthalate, diethylene glycol (DEG-1), diphenyl phthalate, polyesters, styrene oxide, and thiokol, and the inclusion of silicon components in the mixture determines the resistance to heat.

The chemical modification of polymers is carried out by introducing small fragments of a different nature into the composition of macromolecules. Changing the chemical nature of oligomers and hardeners makes it possible to increase the length of the molecular chain of the oligomer and hardener to vary the structure of the internodal sections of the cured system, to modify the end groups of oligomer macromolecules, thereby changing the macroscopic properties of the epoxy polymer. The introduction of reactive additives that are capable of entering into a chemical reaction with the polymer makes it possible to regulate the physical and mechanical properties of epoxides, such as heat resistance, aging resistance, etc., in a wide range [29].

It is known from the literature that dispersed fillers cause a change in almost all properties of liquid and cured epoxy compositions: they increase the viscosity of the binder, and reduce its mobility and viability [30,31,32], as well as affect the crack resistance, mechanical properties, degradation temperature, wear resistance, thermal conductivity, water resistance, and adhesion [33,34,35]. However, the introduction of dispersed fillers into the epoxy binder is accompanied by a deterioration in the water resistance of the material, which limits or makes it impossible to use it in contact with water [36]. Given that cured epoxy resin has a micro-heterogeneous structure [37,38] and its destruction upon contact with water begins with amorphous disordered areas, it is assumed that the introduction of polar fillers into the binder would contribute to the structuring of the cured resin and reduce water absorption; the introduction of non-polar fillers will lead to a disordering of the material and an increase in water absorption [39].

In the study and regulation of the dielectric properties of epoxy oligomers, using various thinners, fillers, curing accelerators, and plasticizers, special attention is paid to the molecular structure of the resulting composites.

The composition (epoxy–bakelite or epoxy–phenolic) has very high dielectric, and especially mechanical, properties, water resistance, and heat resistance. These epoxy compositions are widely used for the production of electrical insulating varnishes and adhesives. Polysulfide resins (thiokols) are also used to cure epoxy resins. The resulting compositions have high elasticity and impact strength, and good dielectric properties are applied to the production of elastic filling compounds. Epoxy–polyester compositions with the introduction of fillers are widely used for the manufacture of casting compounds for the cast insulation of windings of electrical machines, apparatus, and transformers [40]. Plasticizers, solvents, and non-reactive rubbers degrade the properties of epoxy compositions [41]. As active diluents and flexibilizers of ES, aliphatic compounds are widely used as epoxy-containing modifiers based on glycidyl ethers [42].

Due to their low viscosity, they are called active ES thinners, since they are able to replace solvents in the composition of epoxy compositions and copolymerize with ES without releasing by-products. There is a “chemical” modification of the epoxy composition due to the incorporation of flexible fragments into a rigid polymer network and an increase in the molecular weight of the segment [25,43,44]. Epoxy binders have the advantage of low shrinkage and high strength properties. However, scientific and technological progress constantly increases operational requirements, so polymers must be modified. The most promising are composite materials with nanosized fillers—nanocomposites [45,46,47].

The development of new composite materials has expanded the production and use of epoxy resins by several orders of magnitude. Depending on the use, the composition and the amount of filler (solvents, hardeners, stabilizers, plasticizers, and modifiers) are taken into account; thus, the dielectric and physicomechanical properties of cured epoxy resins can vary widely. Depending on the number of epoxy groups, uncured epoxy resins are in a liquid or solid state. They are popular and cover a wide range of applications (from 70 to 90%), such as epoxy–diane resins (diphenylol propane) based on bisphenol A.

The curing agent polyaminoamide is utilized for curing liquid, low-molecular-weight epoxy resins and their compounds. The weight ratio of epoxy resin to curing agent can be adjusted to achieve specific properties in the final cured material. A higher content of the curing agent (above 70% by weight) results in a more elastic and impact-resistant cured resin. However, this comes at the expense of reduced hardness and resistance to high temperatures (attributed to a lower glass transition temperature). This curing agent is suitable for applications requiring cold-cure epoxy resin, where the curing process occurs at room temperature. It is important to note that, according to the product’s safety data sheet, this substance is caustic, may cause skin irritation, and has the potential for sensitization [48].

When epoxy resins are exposed to compounds containing a mobile hydrogen atom, they are able to cure with the formation of three-dimensional infusible and insoluble products with high physical and technical properties. Thus, it is not the epoxy resins themselves that are thermosetting, but their mixtures with hardeners and catalysts. Various substances are used as hardeners for epoxy resins: diamines (hexamethylenediamine, metaphenylenediamine, polyethylenepolyamine), carboxylic acids, or their anhydrides (maleic, phthalic).

Epoxy resins mixed with the above hardeners form thermoset compositions with valuable properties:

- High adhesion to the surface of the material on which they harden;
- High dielectric properties;
- High mechanical strength;
- Good chemical resistance and water resistance;
- Hardening does not emit volatile products and are characterized by low shrinkage (2–2.5%) [40].

Recently, the traditionally used non-reactive modifiers (plasticizers, solvents, and rubbers), which, along with an increase in the elasticity of epoxy materials, lead to a decrease in network density and are able to migrate from the material during operation and increase shrinkage, are being replaced by modifiers that can be embedded in the epoxy matrix. In this case, preference is given to those modifiers that do not lead to the formation of by-products of the reaction and do not increase the number of volatiles released. Such modifiers include, in particular, mono- and polyfunctional low-molecular-weight and oligomeric epoxy compounds, as well as mono- and polyfunctional cyclocarbonates [49].

The study of the phenomenon of heat transfer is very important in the manufacturing of composite materials for protective screens. We consider multilayer plates as radiation shields, which compete with single-layer plates made from the same complex composite materials. Samples in the form of thin plates of epoxy resin grade ED-20 with a filler of soot, graphite, and aluminum powder were studied. Analyzing the results, we can conclude that the introduction of graphite and aluminum powder can significantly increase the effective thermal conductivity of the tested samples. The values of thermal conductivity with carbon black are ambiguous, and to determine the reason for this behavior, an X-ray diffraction study of both the carbon black and graphite that were used is necessary [50].

### 1.4. Dielectric Analysis

Dielectric analysis (DEA) is a method used to monitor the curing process of polymer materials. It involves the measurement of electrical properties, such as dielectric permittivity (or capacitance) and dielectric loss (or conductivity), as a function of time, temperature, or frequency during the curing process. DEA is particularly useful for materials like thermosetting resins, which undergo a chemical transformation during the curing process. It provides a non-destructive and real-time method to monitor and control the curing process, ensuring that the final material possesses the desired properties [51,52,53,54].

In the context of the dielectric analysis (DEA) method, the ionic viscosity parameter and the actual viscosity of a system are related through the dielectric properties of the material being analyzed. Specifically, the ionic viscosity parameter is related to the ion mobility of the material, which in turn can affect its dielectric properties. The actual viscosity of a material refers to its resistance to flow or deformation. It is a measure of the material’s mechanical behavior. The ionic viscosity parameter is, on the other hand, related to ion mobility and electrical behavior; the actual viscosity is related to the material’s mechanical response. In some cases, there may be a correlation between the ionic viscosity parameter and the actual viscosity, especially if the movement of ions significantly impacts the flow behavior of the material [53,54,55].

There are several important parameters of the curing process; among them are the gelation time and curing degree. In brief, the curing degree represents the extent of polymerization or cross-linking that has occurred, while the gelation time is the point in time at which a material starts to transition from a liquid or gel to a solid. Both parameters are significant in various industries where the precise control of material properties is essential [56,57].

The curing process of a thermoset involves a complex series of chemical reactions. Primarily, both gelation and vitrification play pivotal roles [58]. This process commences with the creation and extension of linear chains, which then begin to branch and subsequently cross-link, resulting in the formation of three-dimensional networks in most cases. As the curing progresses, there is an escalation in molecular weight, culminating in various chains forming an extensive network with an infinite molecular weight. This abrupt and irreversible transition from a viscous liquid to a resilient gel is referred to as the “gel point”, which can be defined as the moment when the average molecular weight approaches infinity.

It is worth mentioning that it is also possible to monitor the curing process of a composite material using a multi-channel dielectric analysis (DEA) and potentially correlate it with the thickness of the composite. The thickness of a composite material can influence the curing process. Thicker sections may cure differently than thinner ones due to variations in heat distribution, mass, and heat dissipation. Monitoring multiple channels allows for a more comprehensive understanding of how thickness impacts the curing process. Variations in thickness can lead to non-uniform curing. A multi-channel DEA can detect inhomogeneities in the curing process, providing valuable insights into how different parts of the composite are curing [59,60].

## 2. Experimental Data on Modified ERs and Their Properties

### 2.1. Dielectric Strength Distribution in Modified ER

When modified with various substances, the properties of the polymer matrix (intermolecular interactions) change, affecting the kinetics and mechanisms of molecular processes, which significantly affect the technological and electrical properties of epoxy compounds.

The works [61,62] describe the principle of creating a chemical structure oriented at the molecular level, which makes it possible to create oriented structures along which a heat flux propagates. Based on the chemical structure of bisphenol epoxy resin, it is noted that the thermal conductivity of such oriented materials can reach 10 W/(m K). Oriented structures can also be obtained via the mechanical pressing of elongated fillers, simulating the formation of heat-conducting bridges. In [63], it was shown that when 50 wt% Al_2_O_3_ powder of three different sizes was introduced into the mixture of an epoxy compound (Shell Chemical (Houston, TX, USA)) without chemical modifications of the filler surface, as well as a combination of micro- and nano-sized fillers without changing the total percentage of the filler, the λ values of the epoxy compound increased when moving from the nano to the micro-sized filler. When half of the Al_2_O_3_ particles with a size of 100 μm in the epoxy compound are replaced by particles with a size of 25 nm, there is a significant decrease in the effective thermal conductivity. This may be due to a decrease in the number of large particles of the highly thermally conductive filler necessary to create a heat-conducting cluster [64].

**Figure 2:**
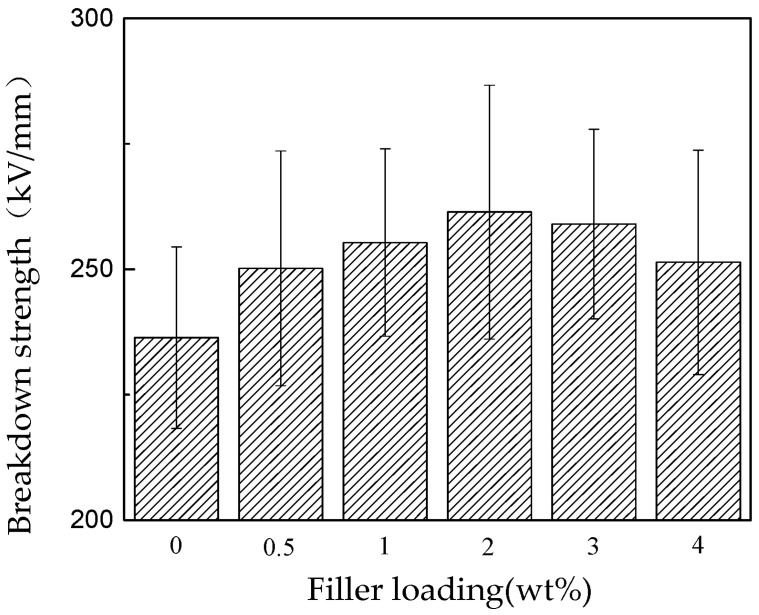
Histogram depicting the breakdown strength of RIP with varying nanoparticle constituents [65].

Figure 2 illustrates the correlation between the DC breakdown strength and nano-SiO_2_ concentration. Initially, the electric breakdown strength of nano-SiO_2_-enhanced RIP (resin-impregnated insulation paper) experiences an ascent with rising content, reaching its zenith at 2 wt%. Subsequently, it diminishes for 3 and 4 wt%. In comparison to unmodified RIP, the maximum DC breakdown strength saw an increment of 10.6% [65].

**Figure 3:**
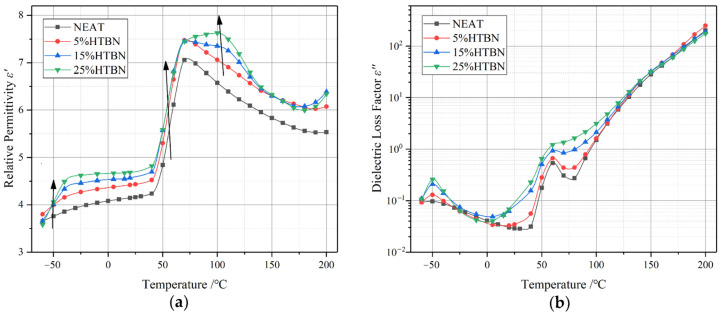
Temperature-dependent spectral properties of epoxy resin with varying proportions of hydroxyl-terminated liquid nitrile rubber (HTBN) at a frequency of 50 Hz: (**a**) relative permittivity; (**b**) dielectric loss factor [66].

Figure 3 displays the temperature-dependent spectral characteristics of the complex permittivity at a frequency of 50 Hz, featuring different levels of liquid rubber content. In Figure 3a, we observe the relative permittivity (ε′), while in Figure 3b, the dielectric loss factor (ε″) is illustrated. Notably, the incorporation of liquid rubber leads to a significant increase in ε′ within the composites.

Moreover, if we follow the arrow in Figure 1a, we notice variations in the dielectric relaxations across distinct temperature ranges: low (from −60 °C to −10 °C), medium (from 0 °C to 90 °C), and high temperatures (from 90 °C to 200 °C). In the low-temperature range, the ε″ initially rises and then declines with the increasing temperature. A notable relaxation peak emerges at −50 °C, representing the secondary transition of the epoxy resin. It is worth noting that composites that have been toughened with liquid rubber exhibit a considerably higher magnitude in the relaxation peak when compared to pure epoxy resin. The positions of the relaxation peak remain consistent across different filler contents, suggesting a superposition effect originating from the orientation of HTBN molecules (HTBN α relaxation) and the secondary transition within the epoxy resin.

There is a patent that describes a method for obtaining an electrically insulating material with excellent insulating properties [64]. The electrically insulating material contains epoxy resin; hardener; elastomeric particles; inorganic particles; and other additive materials. The authors proposed a solution to the problem of reducing the strength and electrical-insulating properties of high-voltage insulation due to the thermal expansion of the epoxy resin and the formation of cracks. The solution lies in the use of dielectric microparticles. The patent discloses a method of mixing together ultrafine particles, a hardener, and a curing accelerator of thermosetting resins. The described method ensures the achievement of excellent electrically insulating, thermally conductive, and mechanical properties of the resulting material. In addition, the proposed inorganic filler may be Al_2_O_3_, TiO_2_, AlN, BN, or a combination thereof. Preference is given to particles with an average size of 500 µm or less.

The patent [67] describes a process for producing an epoxy composite material with a low filler content, high thermal conductivity, and a triple nano/microstructure.

Carbon–polymer composites are widely used in the creation of new structural materials for conductive, heat-absorbing, and shielding coatings. In recent years, special attention has been paid to the study of polymer composite materials based on electrically conductive nanosized fillers, such as carbon nanotubes (CNTs) and nanofibers (CNFs) [68,69].

In [70], the reported dielectric constants for epoxy resins containing varying concentrations of carbon fibers demonstrated an uptick as the carbon fiber content increased. This outcome can be rationalized by the heightened polarity of all the blends resulting from the increased concentration of carbon fibers. This, in turn, leads to an augmentation in the orientation polarization, along with the presence of interfacial polarization. The rise in permittivity as the frequency decreases indicates that the system exhibits interfacial polarization at lower frequencies.

In [71], the results of determining the thermal conductivity and heat capacity of the epoxy material directly in the process of its curing, i.e., with a change in the phase state, are given. An epoxy composition based on epoxy resin and a modified aliphatic polyamine was used as the object of research. The thermophysical properties were determined for different degrees of conversion at room and elevated temperatures. Depending on the degree of conversion, the value of volumetric shrinkage and the values of residual stresses were also evaluated. It was established that during the curing process (i.e., when the degree of conversion changes), the heat capacity decreased by 32% and the thermal conductivity increased by more than 3 times.

During the curing of the epoxy oligomer, its phase state changes and the material initially passes from a liquid to a gel-like state and then to a solid [72,73,74]. The conditions for the transition from a liquid state to a gel state are determined by the values of temperature and the time of gelation [75,76,77].

### 2.2. Thermal Conductivity

**Figure 4:**
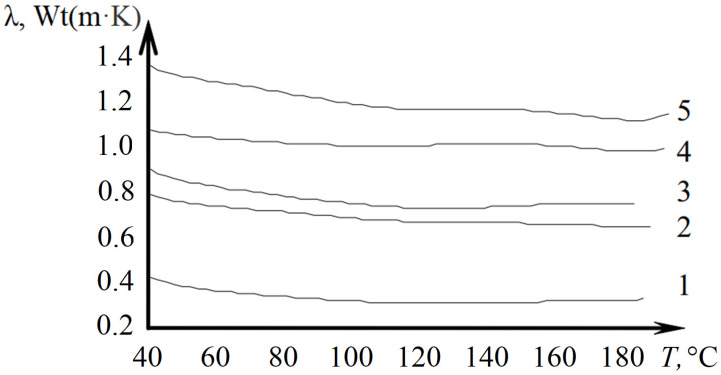
Dependences of thermal conductivity on temperature for materials 1—ED20; 2—ED20 + IrSiO_4_ + TBT; 3—ED + IrSiO_4_; 4—ED + Al_2_O_3_ + TBT; 5—ED20 + Al_2_O_3_ [78].

The study [78] examined the temperature-dependent thermal conductivity of diane resin ED20, both in its pure form and when cured with an aromatic amine (in a 2:1 ratio). Additionally, the investigation included the same polymer with added fillers—aluminum oxide Al_2_O_3_ and iridium orthosilicate IrSiO_4_ (constituting 72.1% of the composition), both in the presence and absence of a modifier, tetrabutoxytitanium (TBT) (see Figure 4).

The incorporation of fillers or modifiers into the polymer has a discernible impact on thermal conductivity. The numerical value λ of the composite material is influenced not only by the quantity of the added component but also by its interaction with the polymer phase.

The results demonstrate that the inclusion of aluminum oxide Al_2_O_3_ significantly augments the thermal conductivity of the composite material, exhibiting a threefold increase across the entire temperature range (40–190 °C) while maintaining a consistent curve profile. Similarly, the use of iridium orthosilicate IrSiO_4_ as a filler doubles the thermal conductivity of the cured epoxy composition within the specified temperature range (40–180 °C). On the other hand, the introduction of tetrabutoxytitanium as a modifier in the composite material composition does not notably alter the temperature-dependent behavior of thermal conductivity.

According to [79], the slight augmentation in the effective thermal conductivity of polymer nanocomposites (PNCs) reinforced with highly conductive nanofillers can be attributed to various factors. Firstly, the presence of carbon nanofillers often leads to agglomeration in bundles within the polymer matrix, driven by the Van der Waals interactions between them. This clustering of fillers may potentially hinder the overall thermal performance of the composite. Secondly, the Kapitza resistance (R_k_) could represent a significant hindrance to heat transfer within PNCs.

### 2.3. Specific Conductivity

The authors of [80] experimentally studied the electrical conductivity of epoxy matrices modified with carbon nanotubes with a continuous current flow during their polymerization at a constant voltage. The research carried out in their work is connected with the study of possible technologies for accelerating the curing of composite materials for the manufacturing of large-sized structures in space conditions. The results obtained can also be used to develop promising technologies for the manufacturing of composite materials with desired electrophysical and mechanical properties by exposing the materials to electric fields of the appropriate configuration during their polymerization.

**Figure 5:**
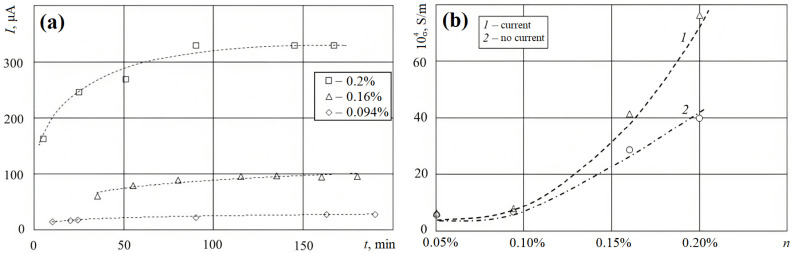
(**a**) Change in current at a constant voltage after adding a hardener (*t* = 0) for different concentrations of CNTs; (**b**) specific conductivity (σ) of the epoxy matrix at various mass concentrations (*n*) of CNTs; 1—samples through which current was passed during polymerization; 2—current was not passed [80].

The investigation of [81], in which a study on the change in current at a constant voltage (28 V) over time from the moment the hardener was added was performed, is shown in Figure 5a, from which it follows that, for the selected cell sizes, the characteristic time of current settling is 100–120 min. Figure 5b shows the specific conductivity (σ) of the epoxy matrix at various mass concentrations (n) of CNTs.

The incorporation of nano-fillers has been widely suggested as a means to improve the dielectric properties of high voltage polymeric insulation, although there are conflicting reports in the literature. According to [81], the potential of silica nanoparticles to prolong the time to failure, particularly by resisting the growth of electrical trees in epoxy resin, was investigated. It was evident that treating the nanoparticles with silane before compounding significantly retarded the tree growth and subsequently extended the time to failure. The growth of trees in needle-plane samples was experimentally assessed in a laboratory, employing loadings of 1, 3, and 5 wt% of nano-fillers. In all instances, the average times to failure showed an increase, but the application of silane treatment to the nanoparticles prior to compounding yielded markedly superior outcomes. Moreover, a distinct initiation period before tree growth was observed in the cases with higher loading levels and silane treatment. For the silane-treated material filled with 5 wt%, the average time to failure was 28 times greater than that of the unfilled resin.

### 2.4. Dependence of Dielectric Constant on Frequency

This study, based on [82], reveals that as the frequency and temperature rise, there is a decrease in breakdown strength. Furthermore, frequency has a significant influence on the reduction in breakdown strength. The application of high voltages introduces complex multi-frequency stresses to the insulation material. The decline in dielectric breakdown strength due to frequency is primarily attributed to heightened partial discharge (PD) activities and dielectric heating, factors that should be taken into account in insulation design. The decrease in the root mean square (RMS) electric fields and the rise in dielectric loss are potential contributors to the diminished breakdown strength of epoxy resin.

**Figure 6:**
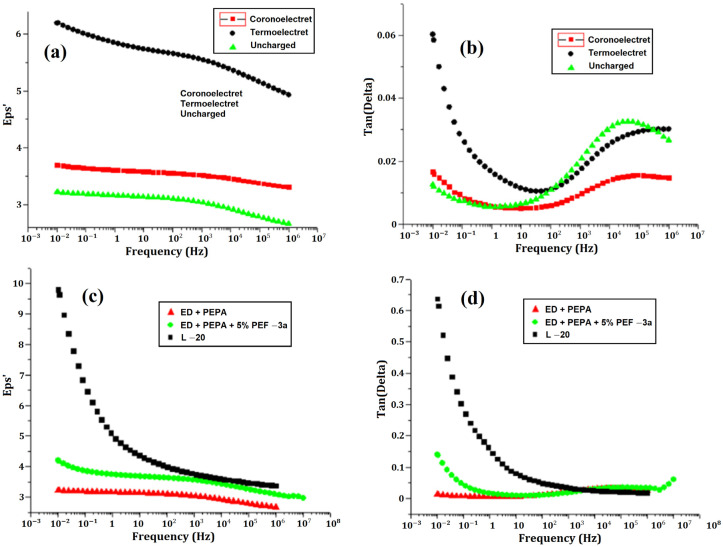
Dependence of dielectric constant (**a**,**c**) and the dielectric loss tangent (**b**,**d**) on frequency for various samples: uncharged sample (1), corona (2), and thermoelectric (3) based on ED-20 epoxy resin cured with PEPA in a stoichiometric ratio, and thermoelectrics based on epoxy resin ED-20, cured with PEPA (1), L-20 (2), and containing 5% wt. PEF-3A, when curing PEPA (3) [83].

The purpose of this work [83] was the study of the permittivity (ε) and the tangent of the dielectric losses (tg δ) of epoxy polymers and electrets based on them. Materials based on the ED-20 epoxy resin, using the oligomeric reactive epoxyurethane modifier PEF-3A, were chosen as the objects of study. To cure the composition, amine-type hardeners PEPA and L-20 were used in a stoichiometric ratio. Figure 6, below, demonstrates the dependencies of the dielectric constant and dielectric loss tangent on frequency for various samples.

To create polymer composites that reduce the impact of electromagnetic fields on electronic components of computer technology and biological objects, it is necessary to obtain materials with specified values of dielectric permittivity, a dielectric loss tangent, and electrical conductivity, which are determined by the operating frequency range of the nanocomposites.

Epoxy binders have the advantage of low shrinkage and high strength characteristics. However, scientific and technological progress constantly increases operational requirements, so polymers must be modified. The most promising are composite materials with nanosized fillers—nanocomposites [45,46,47,84].

### 2.5. Transmittance of Modified ERs

**Figure 7:**
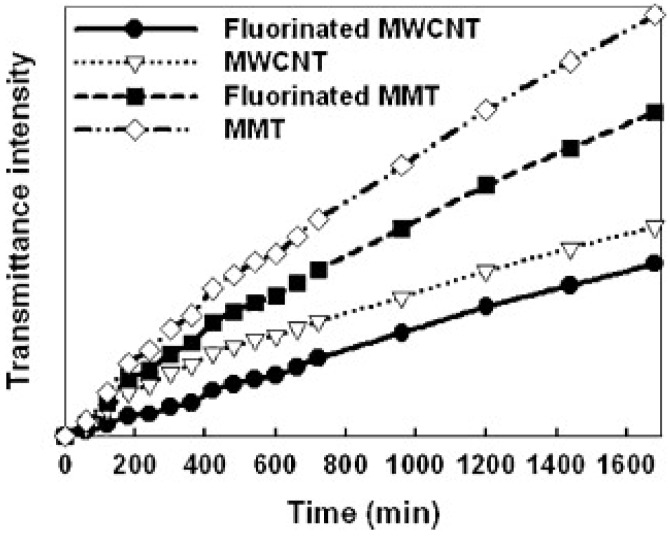
The impact of fluorination on the dispersion of MMT and MWCNTs within epoxy resin. (Figure from [85], permission granted by “Elsevier”).

The research of [85] aimed to assess how the application of fluorination treatment influenced the dispersive stability of montmorillonite (MMT) and multi-walled carbon nanotubes (MWCNTs), as illustrated in Figure 7. In both instances, the transmittance intensity exhibited a reduction following the fluorination treatment, signifying a higher dispersion of particles within the epoxy blend solution. Specifically, the transmittance intensity decreased by approximately 29% for the MMT and 22% for the MWCNTs. This enhancement in dispersion could be linked to the introduction of functional groups onto the surfaces of MMT and MWCNT additives through the fluorination process.

The characteristics of the composite depend on the particle size of the filler. Large filler particles (315–500 microns) are isolated by a dielectric layer, so they have poor contact with each other. The maximum value of the dielectric constant is observed with the introduction of particles of a minimum size of 63–80 µm [86].

### 2.6. Electrical Conductivity of Modified ERs

**Figure 8:**
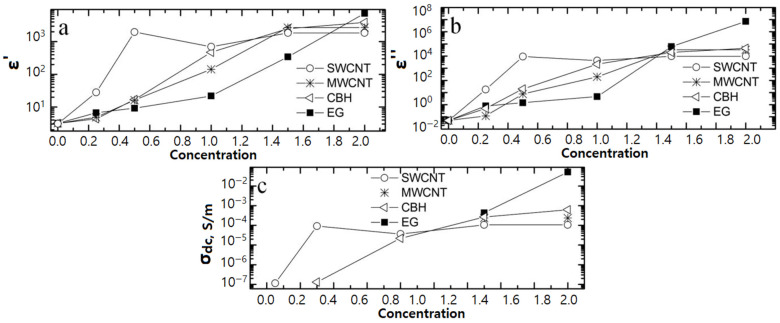
Dependence of ε′, ε″, and σ of epoxy nanocomposites on the concentration of fillers. (**a**) ε′; (**b**) ε″; (**c**) σ_dc_. (Figure from [87], permission granted by “AIP Publishing”).

In [87], the authors investigated the electrical conductivity at direct currents, as well as the dielectric characteristics, of epoxy nanocomposites filled with 37 single-walled carbon nanotubes (SWCNTs), MWCNTs, carbon black (CBH), and graphite (EG) at a frequency of 129 Hz (Figure 8) [84].

Composites containing SWCNTs exhibit their lowest electrical conductivity when operating in the region above the percolation threshold. The rationale behind this observation lies in the fact that SWCNTs, being the smallest in size, consequently offer the highest number of contact regions [86].

**Figure 9:**
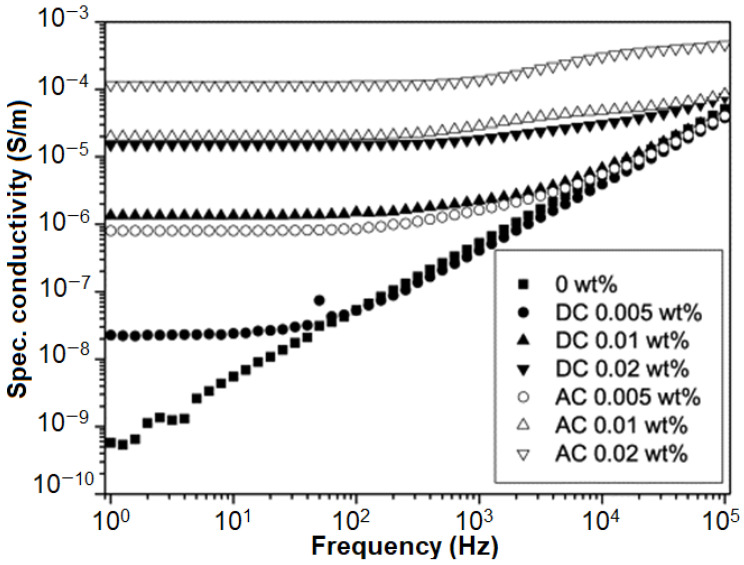
Frequency dependence of the specific conductivity of the epoxy nanocomposite on the concentration of the filler and the method of orientation in the field of direct (DC) and alternating (AC) currents. (Figure from [88], permission granted by “Elsevier”).

In a different study documented in [88], the research delved into the influence of multi-walled nanotube orientation methods on the electrical conductivity of epoxy nanocomposites. The filler was subjected to orientation under both direct current (DC) and alternating current (AC) fields, each with a strength of 100 V/cm (as depicted in Figure 9). The results indicate that exposing the composite to an alternating current field during the curing process leads to a remarkable increase in its electrical conductivity, surpassing that of composites subjected to a direct current, even at equivalent filler concentrations. Hence, it can be inferred that nanotubes demonstrate more efficient orientation when subjected to an alternating field [86].

The incorporation of carbon fillers into the polymer matrix opens up avenues for obtaining materials with diverse properties. Parameters such as the particle shape, aspect ratio, and the evenness of their dispersion within the polymer matrix play pivotal roles in determining both the percolation threshold and the electrical conductivity of the resultant composites. Hence, carbon particles hold significant promise as fillers for crafting composite materials with tailored permittivity values, influenced by the operational frequency range of these nanocomposites [88].

The research of [89] explored the physical and mechanical attributes of epoxy nanocomposites featuring a 0.2 wt% concentration of SWCNTs sourced from OCSIAL, Novosibirsk, Russia.

The study of [90] delved into the curing process of heat-resistant epoxy compositions, enhancing them with functionalized nanotubes CNT-1, CNT-2 (GraNaT, Moscow, Russia), and CNT-3 (BAYER), each possessing specific surface areas of 0.08 m^2^/g, 0.05 m^2^/g, and 0.025 m^2^/g, respectively.

The authors of [91] investigated the impact of low concentrations of functionalized CNTs on the mechanical properties of epoxy nanocomposites. This endeavor yielded a heightened polymer strength and increased limiting deformation upon the incorporation of nanoparticles into a rigid epoxy matrix. Remarkably, the effect that was achieved with low concentrations of functionalized CNTs was akin to the outcomes observed with significantly higher concentrations of pure CNTs. The substantial specific surface area of the filler enables the reduction of the required concentration to attain the desired outcomes [86].

### 2.7. Bending Strength of Modified ERs

**Figure 10:**
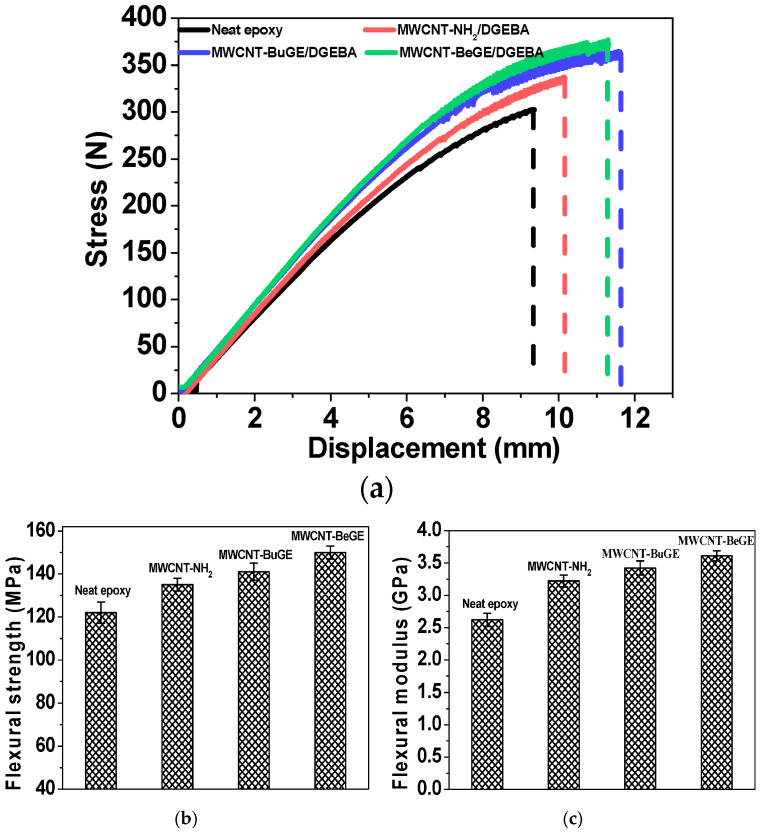
(**a**) Standard curves depicting flexural stress–displacement relationships, (**b**) the associated flexural strength, and (**c**) the flexural modulus for various nanocomposites [92].

The flexural properties of composites composed of multi-walled carbon nanotubes (MWCNTs) and epoxy resin were found to be closely linked to two key factors: the dispersion of the MWCNTs within the matrix and the interactions occurring at the interface between the MWCNTs and the resin matrix [92]. Figure 10a illustrates typical stress-displacement curves for flexural tests conducted on DGEBA/DDM (diglycidyl ether of bisphenol A/diaminodiphenyl methane) reinforced with three different types of MWCNTs: MWCNT-NH2, MWCNT-BuGE (n-butyl glycidylether), and MWCNT-BeGE (benzyl glycidylether). The average flexural strength and modulus are summarized in Figure 10b,c.

The flexural strength and modulus of the pristine resin were measured at 122 MPa and 2.6 GPa, respectively. In comparison, the MWCNT-NH2 reinforced nanocomposites exhibited improved flexural properties, with a strength reaching 135 MPa and a modulus of 3.2 GPa, representing an increase of 11.0% and 23.1% compared to the neat resin.

Conversely, the MWCNT/epoxy nanocomposites treated with surface sizing demonstrated superior flexural characteristics. This enhancement is attributed to the improved dispersion and increased wettability achieved by using epoxy-sizing MWCNTs, as opposed to MWCNT-NH2. The flexural strength and modulus of the MWCNT-BeGE/epoxy nanocomposites could reach 150 MPa and 3.6 GPa, respectively, marking a significant improvement of 22.9% and 37.8% compared to the neat epoxy. Furthermore, when compared with the MWCNT-BuGE/epoxy nanocomposites featuring the same MWCNT content, the MWCNT-BeGE/epoxy nanocomposites displayed a 7.3% increase in strength and a 7.7% increase in modulus, demonstrating that the presence of benzene rings in the surface sizing offered a more effective local load-transfer mechanism between the MWCNTs and the epoxy matrix.

## 3. Discussion

Epoxy polymers are one of the most commonly used polymeric materials in the production of composites. This is due to the properties of epoxy materials, such as high strength, rigidity, chemical resistance, low weight, and a low residual stress in epoxy products due to the low degree of the shrinkage of materials during curing [93,94]. Epoxy polymer materials are insulators. The specific resistance of such materials is >109 ohm∙m [95].

However, for application in some areas, composites based on polymeric materials must have electrical conductivity. One of the important areas for the application of polymer composite materials is the creation of coatings that prevent the accumulation of electrostatic charge (antistatic coatings). In accordance with the international standard IEC 61340-5-1 [96], coatings are divided into conductive (with a resistance R < 106 ohm∙m), antistatic (dissipating static charge and characterized by resistance from 10^6^ ohm∙m to 10^9^ ohm∙m) and insulating (with a resistance of >109 ohm∙m). Another important field in the application of polymer composites is for protection against electromagnetic radiation [97]. To improve the efficiency of shielding electromagnetic radiation, it is necessary to achieve a high conductivity in the protective material. To increase the electrical conductivity of polymer composites, additives of amorphous carbon, carbon fibers, and graphite are used. However, in order to achieve the required coating resistance, it is necessary to introduce a large amount of such additives (>10 wt.%) [98].

The use of nanosized additives makes it possible to achieve the required conductivity of the polymer composite with much lower amounts of the introduced additive. For these purposes, carbon nanotubes are used: multi-walled [99], single-walled [100], and graphene [101]. In [102,103], it is noted that the introduction of small amounts of carbon nanotubes into the composite leads not only to a change in its electrical properties, but also improves the mechanical characteristics of the composite. In addition, carbon-based additives are characterized by a good compatibility with the polymer matrix, light weight, and acceptable electrical parameters [104].

The introduction of graphene particles into the epoxy matrix has little effect on the electrical conductivity and mechanical properties of the composites. The addition of graphene particles to composites containing carbon nanotubes improves their electromagnetic shielding efficiency without significantly affecting their strength [105].

In the study of [106], it was found that composites based on epoxy resin with graphene (10 wt.%) and liquid fillers in situ (base oil SN150 or perfluoropolyether at 10 wt.%) provide low friction and high wear resistance in the form of thin coatings on a steel substrate.

The objective of the study described in reference [105] was to fabricate conductive composites based on epoxy resin that are uniformly filled with multi-walled carbon nanotubes (MWCNTs), graphene particles (GNPs), and MWCNT/GNP mixtures in varying proportions. The research aimed to investigate their electrical and mechanical properties, as well as their effectiveness in shielding electromagnetic radiation within the radio frequency range. The findings revealed that incorporating 2 wt.% of carbon nanotubes into the epoxy matrix increased the electrical conductivity of the composite to 4 S/m. The percolation threshold for such a composite was determined to be 0.013 wt.% MWCNTs. As the electrical conductivity of the composite increased, its ability to shield electromagnetic radiation in the radio frequency range also improved proportionally, reaching 14 dB at 9.25 GHz with the introduction of 2 wt.% MWCNTs. The introduction of graphene particles into the epoxy matrix containing carbon nanotubes had a negligible impact on their conductivity and mechanical properties. When graphene particles were introduced into composites alongside carbon nanotubes, the shielding effectiveness against electromagnetic radiation increased without significantly compromising the composite’s ultimate strength.

In the study [107] a range of functionalized carbon nanotubes (CNTs) was synthesized and their behavior in a colloidal state when combined with epoxy resin and the TETA (triethylenetetramine) hardener was investigated. The discussion centered on the impact of the interfacial interactions between the surface of CNTs and the polymer matrix. The type of functionalization and their unique interactions with polymer chains had notable effects on the physical, thermal, and electrical properties of the composites. For instance, at a 0.5 wt% CNT loading, enhancements in tensile strength were observed as follows: raw CNTs (7.2%), carboxylated CNTs (11.2%), octadecyl amide-functionalized CNTs (11.4%), and hydroxylated CNTs (14.2%). The functionalization of CNTs led to an improvement in the glass transition temperature (Tg) of the epoxy composites, with CNTs-OH demonstrating the highest enhancement (34%) in Tg. Furthermore, the incorporation of CNTs into the polymer matrix resulted in a significant reduction in electrical resistance. The varying re-aggregation behavior of CNTs in the presence of both the polymer and TETA indicated specific interactions between the CNTs and the polymer matrix system. It is worth noting that making a direct comparison with other data is challenging due to the wide range of variables, including the type of epoxy resin, hardener, and CNTs, as well as the degree of functionalization and the processing techniques employed across different studies.

As one of the ways to increase the strength of epoxy resins, the addition of carbon nanomaterials (graphenes and carbon nanotubes) is considered [108,109]. CNTs have significant advantages in this area over traditional dispersive fillers (metal wire, glass fiber, carbon fibers, etc.) due to their ultra-small dimensions (a few to tens of nanometers) and, as a result, being able to fill the micropores of the matrix, as well as due to the possibility of forming chemical bonds with the functional groups of the molecules of the matrix phase [110]. The main task with the introduction of carbon nanotubes as a hardening additive is the separation of tube agglomerates that are formed during synthesis into separate components; therefore, it is necessary to pre-treat CNTs, for example, via oxidative functionalization [111,112].

Scientists from Rice University [113] have developed a remarkable epoxy–graphene composite material that boasts unparalleled compressive strength and remarkably high conductivity. Traditionally, epoxy serves as an insulating material, necessitating the addition of other metals to confer electrical conductivity. However, the researchers at Rice University embarked on a novel approach: they infused the graphene framework with resin. This innovative method yielded a material that was characterized by a compressive strength that breaks records, exceeding that of a pure epoxy bar by a factor of seven. Additionally, the electrical conductivity of this material surged to 41 cm, as reported by Science Daily. It is noteworthy that, prior to this, engineers at the Massachusetts Institute of Technology (MIT) unveiled a self-regenerating material capable of autonomously mending the cracks that form on its surface.

## 4. Conclusions

The complex of the unique physicomechanical and physicochemical properties of epoxy resin allows products based on them to cover a wide range of applications in various fields of electrical and radio engineering. Scientific and technical research that focuses on the development of the most specialized coatings (epoxy fills) that are resistant to highly aggressive environments, improving the properties of epoxy resin as a dielectric (electrical conductivity and insulation strength), and satisfying certain operating conditions is steadily growing.

In conclusion, the resonance of epoxies as vital materials across industries is unmistakable. Their adaptability, versatility, and responsiveness to chemical and electrical factors render them as more than mere compounds—they are dynamic components that shape our technological landscape. By embracing their multifunctionality, researchers and practitioners stand poised to unlock the potential of epoxies in domains ranging from electronics to aerospace, ushering in a future where these remarkable materials continue to be at the forefront of innovation and progress.
